# Supplementary material for: Mediating roles of perceived stigma and mental health literacy in the relationship between school climate and help-seeking behavior in Indonesian adolescents
Source: PLoS One. 2024 May 31;19(5):e0298017. doi: 10.1371/journal.pone.0298017 (PMC11142528; doi:10.1371/journal.pone.0298017)
Supplement: Attachment — Submitted filename: PONE-PONE-D-22-22187.docx [file pone.0298017.s002.docx]

To Editor:
Thank you very much for the opportunity to review your journal. In general, this will be of interest to readers of this journal if the authors add and modify the explanation. I showed several points of request. My conclusion is that if amended, several major points. This paper is worth publishing in your journal.

To Authors:

Thank you for the opportunity to review this manuscript. The authors examined the relationship between the key keywords ``School climate'', ``Mental health literacy'', ``Stigma'', and ``Help-seeking'' in recent mental health challenges in adolescents. Investigating and clarifying these relationships is an essential process in creating an approach to creating a better environment for adolescents in the real world. I would like to ask for some consideration for international researchers and practitioners who are active in this field.

1. Detailed definitions of the keywords ``School climate'', ``Mental health literacy'', ``Stigma'', and ``Help-seeking'' are important to the reader's understanding. For example, the meaning of mental health literacy differs greatly depending on mental well-being, mental ill-health, or focus on treatment and care.

2. Hypotheses about the causal direction of the associations of the variables shown by the authors are not convincing enough in the current manuscript. In particular, a scientific-based theory, etc. should be presented before analyzing why perceived stigma and mental health literacy as mediating roles factors in the relationship between SC and HSB.

3. The results in the abstract require show statistical information.

4. Line 56 (Introduction section)

Are the authors referring to "suicide"? Please add information with several references.

5. I found some similar terms like mental health issues, mental issues, and mental problems. Please provide a clear definition of each.

6. Does Indonesia have a School-based mental health curriculum? Does each school have a staff or care unit taking mental health?

7. Line 114 (Methods section)

The authors described that participants were recruited by convenience sampling. For example, a detailed explanation of the informed consent procedure is required.

8. Line 321 (Descussion section), the direction of Stigma and mental health literacy (Knowledge) is controversial. I would like a careful discussion.
