# Supplementary material for: Mediating roles of perceived stigma and mental health literacy in the relationship between school climate and help-seeking behavior in Indonesian adolescents
Source: PLoS One. 2024 May 31;19(5):e0298017. doi: 10.1371/journal.pone.0298017 (PMC11142528; doi:10.1371/journal.pone.0298017)
Supplement: Attachment — Submitted filename: Reviewer 2.docx [file pone.0298017.s003.docx]

Reviewer:#2

To Editor:
Thank you very much for the opportunity to review your journal. In general, this will be of interest to readers of this journal if the authors add and modify the explanation. I showed several points of request. My conclusion is that if amended, several major points. This paper is worth publishing in your journal.

To Authors:

Thank you for the opportunity to review this manuscript. The authors examined the relationship between the key keywords ``School climate'', ``Mental health literacy'', ``Stigma'', and ``Help-seeking'' in recent mental health challenges in adolescents. Investigating and clarifying these relationships is an essential process in creating an approach to creating a better environment for adolescents in the real world. I would like to ask for some consideration for international researchers and practitioners who are active in this field.

1. Q: Detailed definitions of the keywords ``School climate'', ``Mental health literacy'', ``Stigma'', and ``Help-seeking'' are important to the reader's understanding. For example, the meaning of mental health literacy differs greatly depending on mental well-being, mental ill-health, or focus on treatment and care.

A: Thank you, we really appreciate your comment. We revised some information about definition operational of “school climate”, ``Mental health literacy'', ``Stigma'', and ``Help-seeking'' in the instrument section as below:

“**California School Climate and Safety Survey-Short**

The California school climate and safety survey (CSCSS-SF) was developed by Furlong et al. in 2005 at Centre for School-Based Youth Development California [25]. The CSCSS-SF is a self-report assessment designed to evaluate general school climates and personal safety-related experiences [25]. The CSCSS-SF consisted three major domains, which were school danger, school climate, and victimization [25]. The school climate domain was generated to assess student’s perceptions of school environment including support, respect, safety feeling, and interaction between individuals [25]. In this study, five Likert (from “1=strongly disagree” to “5=strongly agree”) of school climate domain was used to capture school climate among adolescents in Indonesia. School climate domain contains total 13 items, which scored ranging between 13 to 65. The higher score indicating the student feels more positive school climate at school. This study also conducted reliability test and confirmatory analysis of school climate domain. The result showed school climate has acceptable reliability (i.e., 0.77) and good fit indices (ᵡ2 = 125.742, df = 71, p < .001, Tucker–Lewis index [TLI] = 0.93, comparative fit index [CFI] = 0.95, and root mean square error of approximation [RMSEA] = 0.06), indicating school climate domain is a valid and reliable instrument to measure school climate among Indonesian adolescents.

**Mental Health Literacy Scale**

The mental health literacy scale (MHLS) was established in Australia in 2015 by Matt O'Connor and Leanne Casey [26]. MHLS is a comprehensive mental health literacy measure comprising seven attributes: Capability to identify particular conditions; knowledge to gain the information; knowledge about the risk factors and triggers, knowledge of self-treatment; knowledge of available clinical help; and attitude that encourages recognition or effective help-seeking actions [26]. The MHLS contains 19 items with Likert scale responses. The item numbers 1–5 are rated on a four-point Likert scale from 1 (very unlikely/unhelpful) to 4 (very likely/helpful), and item numbers 6–19 are rated on a five-point Likert scale from 1 to 5 (1=strongly disagree/definitely unwilling and 5=strongly agree/definitely willing) [26]. The MHLS total score is generated by summing the scores of all items. Thus, the total score is ranging from 19 to 90 which higher scores indicated higher mental health literacy level. The MHLS was reliable and valid measurement to explore mental health literacy among adolescents, showed by 0.87 for Cronbach’s alpha score and χ2 = 215.042, df = 137, p < 0.0001, goodness-of-fit index = 0.91, TLI = 0.91, CFI = 0.92, RMSEA = 0.05, incremental fit index = 0.93 for construct validity indices which prior analyzed.

**Peer Mental Health Stigmatization Scale**

Peer mental health stigmatization scale (PMHSS) was designed in Ireland to measure adolescent’s related internal stigma and adolescents perception related their society stigma based on the stigma’s components including stereotypes, prejudice, discrimination, and low social status [27]. The items in PMHSS were comprised two major part which negative and positive statements. The negative statement has two domains of stigma agreement (endorsing stigmatization statements on a personal level) and stigma awareness (recognition of the existing societal stigma) [27]. The interpretation of PMHSS was originally separated between negative and positive statement [27]. In this study, the 9 items related negative statement were used to identify the perceived stigma in Indonesian adolescents. The items are rated on a five-point Likert scale from 1 (disagree completely) to 5 (agree completely), and the scores are generated through addition all items, which range 9-45. Higher scores determine higher levels of perceived stigma. Based on our analysis, PMHSS was reliable and valid to be used in Indonesian adolescents. The reliability of PMHSS in Indonesian version was 0.78 and the construct validity indices were χ2 = 38.90 (χ2/df = 1.62), TLI = 0.93, CFI = 0.95, RMSEA = 0.05, SRMR = 0.03.

**General Help-Seeking Questionnaire**

The general help-seeking questionnaire (GHSQ) was created to investigate the help-seeking intentions of any sources and issues [28]. The GHSQ has been used in several studies and found there was a positive association between the general help-seeking and aspects of emotional competence [29, 30] in both prospective and retrospective behavior of help-seeking [31]. This help-seeking intention questionnaire includes formal and informal sources of help options aside from “self” or “no one” (e.g., counselor, peers, relatives, and no one). The GHSQ requests participants to rate their help-seeking intentions on a seven-point scale (1 = extremely unlikely, 7 = extremely likely) to each problem type for each source of help option including “no one.” Higher scores indicate higher intentions to seek help [28]. The Cronbach’s alpha of GHSQ was 0.86.

” [Line 141-200]

2. Q: Hypotheses about the causal direction of the associations of the variables shown by the authors are not convincing enough in the current manuscript. In particular, a scientific-based theory, etc. should be presented before analyzing why perceived stigma and mental health literacy as mediating roles factors in the relationship between SC and HSB.

A: Thank you for your comment. We added some information in the introduction as below.

“According to reasoned action theory, people's behavior is expected to be rational, consistent, and frequently automatic, based on their beliefs regarding the behavior they are performing [12]. There are three factors affected people’s behavior [12]. First, background factors related information such as knowledge. Second, people’s beliefs including behavioral belief such as stigma that followed by attitude toward the behavior. Third, the behavior can be directly influenced by factors of actual control, such as the environment. The existence of environmental limitations can hinder individuals from engaging in specific behavior including help-seeking behavior [12]. Based on studies, adolescents in Asia spend most of their time at school, which makes school has pivotal roles in maintaining adolescents mental well-being [13-15]. In this regard, the school climate was pronounced highly associated with help-seeking behavior. The supportive school environment from teachers, peers, and safety environment increased the willingness of adolescents to express their concern and accessing help [16-18]. In addition, positive school climate (i.e., safety, engagement and environment aspects) engaged adolescents to increase literacy and decrease stigma about mental health problem [19]. Moreover, prior studies suggested that perceived or internal stigma on mental health problem would inhibit adolescents to seek for help [9, 20, 21]. On the other hand, higher mental health literacy level encouraged adolescents seeking for help [22]. Based on aforementioned explanation, the association among environment (school climate), knowledge (mental health literacy), behavioral belief (perceived stigma), and behavior (help-seeking behavior) need to be explored further (see Fig1).” [Line 76-95]

Mental Health Literacy

Perceived Stigma

Help-Seeking Behavior

School Climate

**Fig 1.** Hypothesis Framework Based on Reasoned Action Model and Related Literatures

3. Q: The results in the abstract require show statistical information.

A: Thank you very much, we revised as below:

“Findings indicated that ethnicity, family income, and father’s educational level were significantly associated (p < 0.05) with help-seeking behavior in Indonesian adolescents. Furthermore, perceived stigma and mental health literacy sequentially showed partial mediating roles in the relationship between school climate and help-seeking behavior (indirect effect: 0.004; 95% CI: 0.001, 0.010). Our mediating model indicated that a high level of school climate was associated with low perceived stigma (b = −0.11, p < 0.001) and high mental health literacy (b = 0.28, p < 0.001) and higher help-seeking behavior (b = 0.14, p < 0.001).” [Line 36-42]

4. Q: Line 56 (Introduction section)

Are the authors referring to "suicide"? Please add information with several references.

A: Thank you for your concern, We updated the information with the reference as below:

“Mental health problems among adolescents contributed to suicide and self-harm that ranking as the fourth leading cause of mortality in the world [8] ” [Line 71-73]

5. Q: I found some similar terms like mental health issues, mental issues, and mental problems. Please provide a clear definition of each.

A: Thank you for your comment, the terms of mental health issues, mental issues, and mental problems in study refer to mental health problems. According to Mental Health Foundation, mental health problems are difficult experiences that make it harder for us to get on with our lives. Since, we enrolled healthy participants, therefore, we used the term “mental health problems”. Additionally, to make our manuscript more consistent, we revise the aforementioned term (i.e., mental health issues, mental issues, and mental problems) into mental health problems.

Reference: <https://www.mentalhealth.org.uk/explore-mental-health/about-mental-health-problems>

6. Q: Does Indonesia have a School-based mental health curriculum? Does each school have a staff or care unit taking mental health?

A: Thank you for your question. According to Ministry of Education, Culture, Research, and Technology (policy number 111, year 2014), every school in Indonesia has a section of guidance and counseling that refers to a process of providing assistance, support, and guidance to students in various aspects of their personal, academic, and career development. Meanwhile, instead of psychiatrist, the teachers who were major in guidance and counseling are responsible for this service.

Reference: <https://jdih.kemdikbud.go.id/sjdih/siperpu/dokumen/salinan/Permendikbud%20Nomor%20111%20Tahun%202014.pdf>

We added some information in the introduction part as below:

“In Indonesia, the Ministry of Education, Culture, Research, and Technology (KEMENDIKBUD) implemented Policy Number 111 in 2014 in response to the growing concerns about mental health issues among adolescents [5].” [Line 57-60]

7. Line 114 (Methods section)

Q: The authors described that participants were recruited by convenience sampling. For example, a detailed explanation of the informed consent procedure is required.

A: Thank you very much. We revised and added some information related procedure in the method part. The paragraph as below:

“This study enrolled the participants from four public senior high schools (i.e., SMAN 1 Gading, SMAN 1 Kraksaan, SMAN 1 Probolinggo, SMAN 1 Gending), which located in East Java, Indonesia. The convenience sampling method was performed to collect the participants from July to August 2019. The inclusion criteria were students who completed the informed consent form and obtained permission from their parents or guardian; who could write and read Indonesian; who were aged 16–19 years; and who were in grade 10–12th.

Students who were undergoing treatment for mental health disorders or were absent from school during data collection were excluded from the study. The sample size calculation was conducted using G*Power software version 3.1.9.4 [23]. The sample size was calculated under statical test of multiple linear regression analysis with 12 predictors, including demographic variables, school climate, perceived stigma, and mental health literacy. The effect size of 0.15 [24], Cronbach’s alpha of 0.05, and a power of 0.95 were inputted. the sample size was calculated equals to 184 participants. After considering the dropout rate of 20%, this study at least recruited 184+37= 221 participants. However, the total of 760 participants from four public senior high schools were enrolled in this study.

In this cross-sectional study, the correlation among school climate, perceived stigma, mental health literacy, and help-seeking behavior were explored. Prior to data collection of aforementioned variables, the students of four public senior high schools (i.e., SMAN 1 Gading, SMAN 1 Kraksaan, SMAN 1 Probolinggo, SMAN 1 Gending) were required to read and complete the informed consent, as well as, obtained the permission from their parents or guardian by signing informed consent letter for parent or guardian. Three days after, the collected the signed informed consents and questionnaires about demographic variables, California school climate and safety survey-short form (school climates), mental health literacy scale (mental health literacy), peer mental health stigmatization scale (perceived stigma), and general help-seeking questionnaire (help-seeking behavior). The total duration of completing all questionnaires was 40 minutes with 1 time of 5 minutes break. The participants were allowed to quit from the study in every times if they were not willing to complete all questionnaires. The researcher explained that this study was anonymous and would not affecting their school performance. In addition, a gift would be given to the participants who completed all questionnaires.” [Line 106-138]

8. Q: Line 321 (Descussion section), the direction of Stigma and mental health literacy (Knowledge) is controversial. I would like a careful discussion.

A: Thank you very much for your concern, we revised and added some information related the direction of stigma and mental health literacy. The paragraph as below:

“ Some studies have shown that perceived stigma predicted help-seeking behavior [49, 50], which is different from our study’s finding. In this study, perceived stigma was not significantly associated with help-seeking behavior. In line with this finding, a previous study by mentioned that perceived stigma was not found to be associated with less willingness to help-seeking, especially in delivery their mental health problems to the helper [51]. Furthermore, [51] noted that in the rural population, the help seeking was not associated with perceived stigma due to in rural populations tend to cope with their mental health problems by themselves [51]. The present study enrolled part of its participants from rural areas, which may have influenced the correlation between perceived stigma and help-seeking behavior in this study due to tend to cope up with their mental health problems by themselves. In addition, family income significantly associated with help-seeking behavior and the highest percentage of participants in our study was from low-income families, that might be the obstacle to seek help for their mental health problem. Prior longitudinal study described no significant association between perceived stigma and help-seeking behavior, its due to some variables that may confound the relationship was not observed [20]. It was consistent with our finding, perceived stigma significantly associated with help-seeking behavior after simultaneously analyzed in our mediation model. We assumed some factors such as family income affected the association of perceived stigma and help-seeking behavior. Another point of view, a study mentioned that personal stigma was more prominent barrier compared to perceived stigma for young people seeking help [52]. Young people who confident about their treatment or behavior might be less concern of the attitude of most people (public stigma) such friends and family.” [Line 345-365]

“In contrast with prior study, social and self-stigma was described to have significantly partial mediating effect on relationship mental health literacy and help-seeking attitudes [57]. Higher mental health literacy indicate an adequate knowledge and beliefs of young people to aid to destigmatize and manage mental health problems event [57]. This different finding was assumed due to vice versa relationship of perceived stigma and mental health literacy. These two variables was important to determine help-seeking behavior. The recognition of mental health problems could be achieved by adequate mental health literacy and the stigma could affect the decision making to take the action for seeking-help [58]. Reversely, large number of stigma from their society intend to inhibit adequate mental health literacy then followed by unidentified mental health problem and ended to inadequate help-seeking.” [Line 382-392]
